# Design of a Novel Auxiliary Diagnostic Test for the Determination of Authenticity of Tequila 100% Agave Silver Class Based on Chemometrics Analysis of the Isotopic Fingerprint of the Beverage

**DOI:** 10.3390/foods12132605

**Published:** 2023-07-05

**Authors:** Rocío Fonseca-Aguiñaga, Uriel E. Navarro-Arteaga, Martin Muñoz-Sánchez, Humberto Gómez-Ruiz, Walter M. Warren-Vega, Luis A. Romero-Cano

**Affiliations:** 1 Grupo de Investigación en Materiales y Fenómenos de Superficie, Departamento de Biotecnológicas y Ambientales, Universidad Autónoma de Guadalajara, Av. Patria 1201, Zapopan 45129, Mexico; rfonseca@crt.org.mx (R.F.-A.); uriel.navarro@edu.uag.mx (U.E.N.-A.); wwarrenv12@gmail.com (W.M.W.-V.); 2 Laboratorio de Isotopía, Consejo Regulador del Tequila A. C., Av. Patria 723, Zapopan 45030, Mexico; 3 Consejo Regulador del Tequila A. C., Av. Patria 723, Zapopan 45030, Mexico; mmunoz@crt.org.mx; 4 Departamento de Química Analítica, Facultad de Química, Universidad Nacional Autónoma de México, Ciudad de México 04510, Mexico; hgomez@unam.mx

**Keywords:** isotopic fingerprint of Tequila, authenticity of Mexican alcoholic beverages, agave, appellation of origin

## Abstract

The present research shows a robust isotopic ratio characterization of Carbon-13 (δ^13^C_VPDB_) in congeneric compounds such as methanol, n-propanol, isoamyl alcohol, ethyl lactate, ethyl acetate, ethanol, and acetaldehyde in representative samples (*n* = 69) of Tequila 100% agave silver class (TSC), employing gas chromatography/combustion/isotope-ratio mass spectrometry (GC/C/IRMS). From the information obtained, the construction of a radial plot attributable to the isotopic fingerprint of TSC was achieved. With this information, a diagnostic test was designed to determine the authenticity of TSC, comparing alcoholic beverages from other agave species as non-authentic samples. The sensitivity of the test was 94.2%; the specificity was 83.3%. Additionally, non-authentic samples were analyzed that meet all the criteria established in the regulations. The results obtained show that the GC/C/IRMS analytical technique and designed diagnostic test are useful as auxiliary parameters to determine the authenticity of the beverage, thus managing to determine the adulteration or falsification of the product.

## 1. Introduction

Tequila is an alcoholic beverage prepared from the distillation of musts of *Agave tequilana* Weber blue variety [1]. According to historical data from the Tequila Regulatory Council (CRT for its acronym in Spanish), sales records are broken annually in Mexico and the world, evidencing the importance of the beverage in the markets. Because of the success of the beverage, unfair practices have been detected where the presence of adulterants or counterfeit beverages are highlighted, as has happened with other successful alcoholic beverages [2,3]. According to recent studies, it has been appreciated that the realization of these illegal practices has proved to be one of the great obstacles to covering the supply chain of Tequila, because they have caused health problems for the consumer; in addition, there is an impact on the image, which represents an economic loss in terms of beverage sales [4,5].

With the purpose of safeguarding the quality and authenticity of Tequila and its production process, a permanent inspection process is carried out by the CRT to ensure the agave–Tequila production chain. With the objective of safeguarding the Tequila beverage, several research groups have focused on implementing analytical techniques that allow for auxiliary methods in quality controls, which can be mainly classified into three groups: chromatographic [6,7,8,9,10,11], spectroscopic [12,13,14,15,16,17], and electronic [18,19]. Some of these have already been implemented in other alcoholic beverages such as wine, for example, the fractionation of natural isotopes of a specific site studied by nuclear magnetic resonance (SNIF-NMR) [20,21].

Nevertheless, the use of GC/C/IRMS stands out since it has been shown that it is possible to determine robust analytical parameters, which can be helpful as auxiliary parameters for inspection processes [9,22,23,24]. However, there is still a need to strengthen the technique to be able to discriminate Tequila from other alcoholic beverages prepared from other agave species (Mezcal, Sotol, Bacanora, and Raicilla), since these agave species belong also to the Crassulacean Acid Metabolism and share similarities in their morphological aspects and chemical compounds that enrich product such as fructans [25,26], but according to the specifications established in the Official Mexican Standard NOM-006-SCFI-2012 the only plant used for Tequila production must belong to the agave species of *Agave tequilana* Weber blue variety. For this reason, it is important to establish a diagnostic test capable of distinguishing and discriminating between Tequila 100% agave silver class, non-authentic beverages, and alcoholic beverages produced from other species of agave different from *tequilana* Weber blue variety in order to ensure the authenticity of the beverage.

Due to the above, the present research proposes the use of gas chromatography coupled with isotopic ratio mass spectrometry to determine δ^13^C_VPDB_ values of the molecules that are congeneric compounds in the beverage as an auxiliary method to determine the authenticity of the beverage (methanol, n-propanol, isoamyl alcohol, ethyl lactate, ethyl acetate, ethanol, and acetaldehyde). For this purpose, the characterization of representative samples of Tequila 100% agave silver class produced in the territory that grants the Appellation of Origin of Tequila (DOT for its acronym in Spanish) in the Mexican Official Standard NOM-006-SCFI-2012 will be carried out. From the experimental data, radar plots (isotopic fingerprint) were built and contrasted against representative samples of Mezcal, Sotol, Bacanora, and Raicilla.

## 2. Materials and Methods

### 2.1. Samples

We had the objective of analyzing samples of Tequila 100% agave silver class from all producers in the Appellation of Origin of Tequila. Stratified sampling was used to divide the number of samples based on the active Tequila producers. We had the support of CRT in requesting random samples of Tequila 100% agave beverage from different Tequila companies (*n* = 69) located in Guanajuato, Michoacan, and different regions of Jalisco state (Valles, Altos Sur, Cienega, and Centro), as shown in Table 1. In addition, it is important to highlight the economic impact of other alcoholic beverages obtained from another species of *Agave tequilana* Weber blue variety. Therefore, to assess the sensitivity and specificity of the isotopic fingerprint, an analysis was performed with: Mezcal (22 samples), Bacanora (8 samples), Sotol (2 samples), and Raicilla (4 samples). Different producers provided all the beverages to the Isotope Laboratory of the CRT for research purposes.

### 2.2. Determination of the Isotopic Ratio of Carbon 13 in Congeners Present in Tequila 100% Agave Silver Class

Firstly, to determine the isotopic ratios, a gas chromatograph (Thermo Scientific, Waltham, MA, USA), model Trace 1310 was used. To carry out the separation of compounds, a DB-Wax column (stationary phase of polyethylene glycol, 60 m long, 0.25 mm internal diameter and 0.25 μm thick) was used. The autosampler was set up using a 10 μL syringe with an injection volume of 1 μL in split mode with a split ratio of 1:40. The temperature at the injection port was 200 °C. During the experimental run, a carrier gas flow of 1.2 mL min^−1^ was maintained. After that the eluted compounds enter the combustion cell and the gases enter the IRMS in a continuous process using a spectrometer Thermo Scientific Model Delta V Plus. The operating conditions of the GC/C/IRMS analyses are presented in Supplementary Table S1.

In Supplementary Material, there can be found the information with respect to reagents and gases used in the systems for analytical measurements. Results were expressed in parts per mil (‰) by a normalization of the results obtained from the reference gas previously standardized with respect to an international reference VPDB (Vienna Pee Dee Belemnite). The relative difference of isotope ratios (isotope-delta values) was obtained by Equation (1).

(1) $${\delta (}^{i/j}E)=\delta^{i/j}E=\frac{{\;}^{i/j}R_{p}-{\;}^{i/j}R_{\mathrm{ref}}}{{\;}^{i/j}R_{\mathrm{ref}}}\;$$

where *^i^E* denotes the higher (superscript *i*) and *^j^E* the lower (superscript *j*) atomic mass number of element *E*. According to the previous reference, the values for *δ^13^C_VPDB_* were obtained from Equation (2):

(2) $$\delta^{13}C_{VPDB}=\left[\frac{{\left({\;}^{13}C/{\;}^{12}C\right)}_{sample}}{{\left({\;}^{13}C/{\;}^{12}C\right)}_{standard\;VPDB}}-1\right]$$

To provide certainty to the measurements, a strict quality control is maintained within the laboratory to guarantee the reliability of the results.

All samples were analyzed by triplicate, and it was established that standard deviation of the reference was ≤0.2‰ and for the samples ≤ 1.8‰. Likewise, the value of the linear correlation for the normalization curve must be greater than 0.99.

### 2.3. Quantitative Analysis of Problem Samples

Based on the official methodology established in the NMX-V-005-NORMEX-2018, congener compounds were analyzed (aldehydes, esters, higher alcohols, and methanol). An Agilent 7890 B gas chromatograph (Agilent Technologies, Boston, MA, USA) with a flame ionization detector and automated sampler with capillary injection was used. An Agilent J&W DB-WAX UI 60 m by 0.25 mm and 0.25 µm column was used with a backflush system. The furnace was programmed for 3 min with a temperature ramp starting at 37 °C. After that, a constant increase of 3 °C min^−1^ occurred until a temperature of 80 °C was reached and held for 1 min. Then, increases of 15 °C min^−1^ occurred until 160 °C was reached and held for 3 min. Then, an increase of 50 °C min^−1^ occurred until 220 °C was reached, and it was kept constant for 4 min. In all the samples analyzed, nitrogen was used as a gas carrier at a volumetric flow rate of 1.3 mL min^−1^ and was injected into a sample volume of 1.0 µL in a split ratio of 49:1 (split mode). Lastly, the adjustment was set to 220 °C for the injection and detection temperatures. A typical GC/C/IRMS chromatogram of a sample of Tequila 100% agave silver class is presented in Supplementary Figure S1.

### 2.4. Statistical Analysis

STATISTICA 10.0 software (StatSoft, Palo Alto, CA, USA) was used to analyze the experimental data. The descriptive statistics analysis was performed by determining the mean, standard deviation, quartiles (lower and higher), median, coefficient of variation, range, and minimum and maximum values recorded for δ^13^C_VPDB_ of each congener studied. Additionally, a one-way analysis of variance (ANOVA) was employed to determine the existence of statistically significant differences between the means of the groups (Tequila 100% agave silver class, Mezcal, Bacanora, Raicilla, and Sotol) using a significance level of 95%.

The auxiliar diagnostic test to determine the authenticity of the beverage was performed by determining the δ^13^C_VPDB_ of each of the congeners studied (methanol, n-propanol, isoamyl alcohol, ethyl lactate, ethyl acetate, ethanol, and acetaldehyde). The values obtained were contrasted against the maximum and minimum limits established by the standard deviation of each test. In case the value was within the established limits, a value associated with the coefficient of variation (C.V.) was assigned. On the contrary case, the assigned value corresponded to 1-C.V. Once the value corresponding to each of the congeners was assigned, a final score was obtained for the sample by multiplying all the values. Finally, this score is used to determine if the evaluated samples correspond (positive diagnostic) or not (negative diagnostic) to Tequila 100% agave silver class, which was evaluated using the Receiver Operating Characteristic (ROC curve) statistical method.

## 3. Results

### 3.1. Statistical Analysis

**Figure 1:**
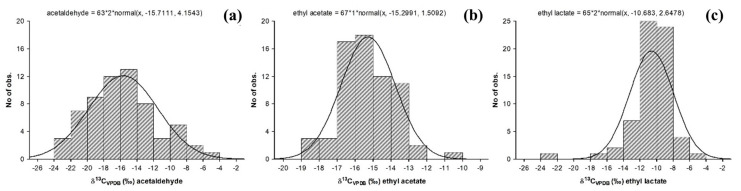
δ^13^C_VPDB_ (‰) in molecules of aldehydes and esters present in samples of Tequila 100% agave silver class: (**a**) acetaldehyde, (**b**) ethyl acetate and (**c**) ethyl lactate.

**Figure 2:**
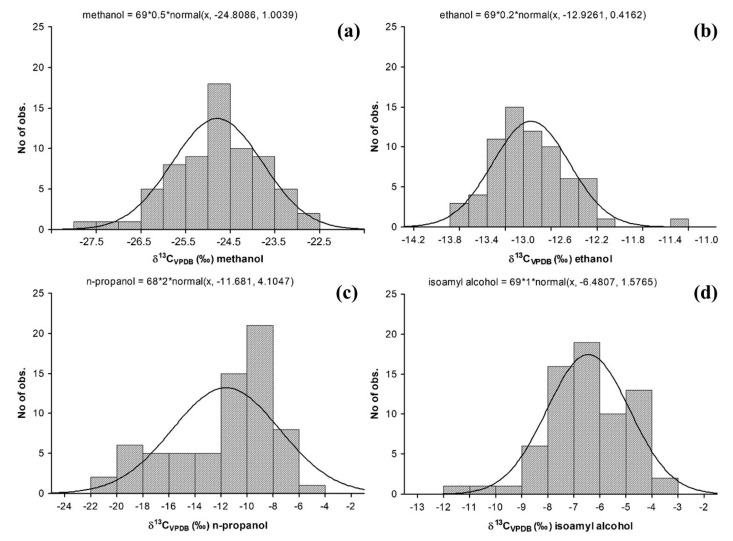
δ^13^C_VPDB_ (‰) in alcohol molecules present in samples of Tequila 100% agave silver class: (**a**) methanol, (**b**) ethanol, (**c**) n-propanol and (**d**) isoamyl alcohol.

Table 2 shows the statistical analysis realized in the experimental data obtained from the Tequila 100% agave silver class samples. It can be observed that the data for acetaldehyde (Figure 1a), ethyl acetate (Figure 1b), methanol (Figure 2a), and ethanol (Figure 2b), have a behavior that fits to a normal distribution, with values of a coefficient of variation less than 10% with a mean value at −15.7‰, −15.3‰, −24.8‰ and −12.9‰, respectively. Among them, the values obtained for ethanol and methanol stand out because their values can be associated with the agave plant used in beverage production, so these parameters can be considered to be broadly representative of the universe of samples studied.

The values obtained of δ^13^C_VPDB_ in the ethanol complements satisfactorily previously reported studies, where it has been shown that the values are representative of the plant used as a source of sugar in alcoholic fermentation (agave), which corresponds to a plant of the CAM (Crasulacean Acid Metabolism) group that represents with a particular photosynthesis process for CO_2_ atmospheric fixation due to a combination from Calvin and Hatch Slack cycles [23,24].

To the above, the presence of this congener in Tequila is considered a priority, within the range of δ^13^C_VPDB_ reported here, to declare a Tequila 100% agave silver class as an authentic beverage, so its criteria within the evaluation of authenticity must be stricter for the rest of the congeneric compounds.

Additionally, the values of δ^13^C_VPDB_ in the methanol can be attributed to the agave plant species used. Supplementary Figure S3 provides information in this regard, it is observed that there are significant differences (*p* < 0.05) between Tequila (−24.8‰ in average), Sotol (−35.14‰ in average), Mezcal (−23.19‰ in average) and Bacanora (−27.7‰ in average). It is proposed that during the hydrolysis stage the generation of methoxy groups (-O-CH_3_) occurs, which are the precursor of methanol, due to the thermal treatment of agave pectins. The methoxy groups are strongly bound to a vegetal matrix (R), during the thermal treatment demethoxylation, and consequently methanol formation occurs, which remains in the liquid or is transferred to the vapor phase (Equation (3)):

(3) $$R-O-CH_{3}\overset{∆}{\to }R+CH_{3}-OH_{\left(solution\right)}+CH_{3}-OH_{\left(steam\right)}$$

The most negative of *δ^13^C_VPDB_* values may indicate that methoxylated groups are present in diverse forms inside the vegetal structure (dependent on agave species), some of which are recalcitrant to thermal treatment [27]. Supplementary Table S2 presents the different species of agaves used to produce each of the beverages. In summary, Mezcal is produced from approximately 14 different types of agaves, corresponding to all the species of agave found within the territory of the Mezcal appellation of origin, which covers 13 states [28]. Sotol is produced from an endemic agave called *Dasylirion wheeleri*, native to the states of Durango, Chihuahua, and Coahuila [29]. Bacanora is a beverage native to the state of Sonora and produced from the *Agave angustifolia* Haw [30]. Finally, the Raicilla is native to Jalisco state, prepared from any agave grown within its appellation of origin area, and unlike Tequila, it must be made from 100% agave, and additional distillations with a variety of ingredients are allowed for the presence of various flavors, which can be added directly [31].

On the other hand, δ^13^C_VPDB_ values for n-propanol (Figure 2c), isoamyl alcohol (Figure 2d), and ethyl lactate (Figure 1c) tend to have a normal distribution with a value that has a positive trend, with centered means at −11.7‰, −6.5‰ and −10.7‰, respectively. Consequently, these compounds have higher values in their variation coefficients (>20%), and this effect its related to Tequila 100% agave production processes. It is proposed that these variations could be due to the fermentation and distillation stages, due to the fact that each company possesses yeast strains and specific operational parameters to grant unique organoleptic properties to their products [32,33,34,35,36,37]. These differences can also be observed when comparing the values of Tequila against alcoholic beverages produced from other agave species. Unlike the production of Tequila, the production processes of Mezcal, Sotol, Raicilla, and Bacanora follow artisanal practices, highlighting the stages of cooking, fermentation, and distillation [33,38,39,40].

For the cooking stage, the agave heads are placed inside a hole dug in the ground, where they are cooked (hydrolysis) for approximately 48 h. In artisanal processes, the fermentation stage is carried out without a strict control since it happens spontaneously in casks exposed to the environment for 4 to 5 days, with low ethanol yields and a non-physicochemical enriched product of volatile organic compounds. Although this process is also used in some Tequila companies, this practice can be considered mostly used for beverages produced from other agave species than *tequilana* Weber blue variety [41]. Finally, the distillation stage is carried out in batches, leaving this process to the discretion of the supervisor (commonly called “vinatero”) [42], which are chosen by visual contrast when it is observed on the surface bubbles that can burst. Due to the above, the values of δ^13^C_VPDB_ in the molecules of n-propanol, isoamyl alcohol, and ethyl lactate present significant differences with respect to Tequila. This concludes that variations of these congeners must be reviewed with less strict criteria than those established for ethanol, methanol, and ethyl acetate, as detailed in the next section.

### 3.2. Linear Discriminant Analysis

To determine if it is possible to classify samples belonging to different groups (Tequila, Mezcal, Sotol, Bacanora and Raicilla), a Fisher Linear Discriminant Analysis was performed. During the analysis, 30 samples were excluded due to the loss of at least one discriminant variable, that is, the presence of all the congeners studied was not detected in the excluded samples. Due to the above, the analysis was carried out with 75 samples.

Based on the information obtained, the combined intragroup correlations between the variables and the typified canonical discriminant functions were obtained. Based on this information, the congeners that have the greatest weight to classify each beverage were determined (Table 3). In the case of Tequila, the congeners that have a higher absolute correlation are methanol (0.415) and ethyl acetate (0.717), while in the case of Mezcal they are ethanol (0.470) and isoamyl alcohol (0.635); finally, for Bacanora they are isoamyl alcohol (0.633) and n-propanol (0.760). It is important to highlight that since there were not enough cases of Sotol and Raicilla, they could not be evaluated.

**Figure 3:**
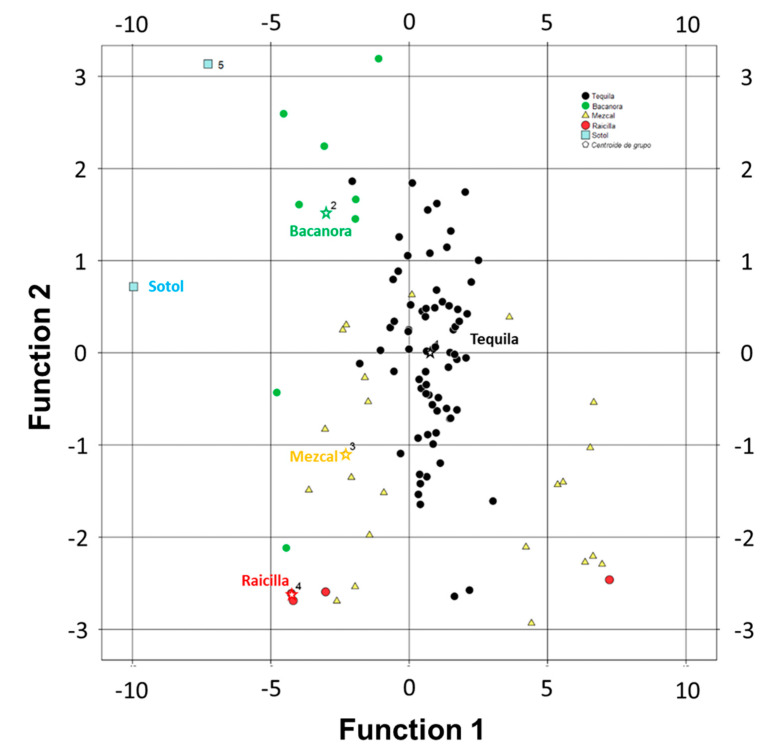
Graphical representation of the experimental data used in the discriminant function analysis. Where: ★ corresponds to the group average and thenumbers are referred to the group.

The graphic representation of the classification of the analyzed samples is presented in Figure 3. It is observed that the discriminant functions obtained are not capable of classifying the analyzed beverages since in the case of Bacanora and Mezcal the analyzed samples can be classified as 100% Tequila agave, without being. Therefore, the use of new chemometric strategies becomes essential to determine the authenticity of the beverage.

**Figure 4:**
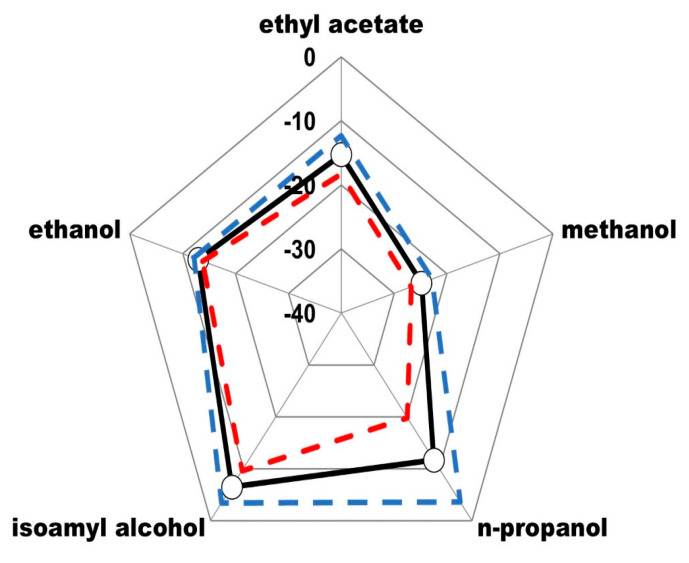
Isotopic fingerprint (Radar plot) of δ^13^C_VPDB_ (‰) mean values for Tequila 100% agave silver class: ○ average, --- average + 2s, --- average − 2s.

Based on the previous chemometric analysis, it was defined that the molecules of ethyl acetate, methanol, n-propanol, isoamyl alcohol and ethanol correspond to the congeners that have the greatest weight to classify each beverage. Considering the above, the radar plot presented in Figure 4 was constructed, representing the isotopic fingerprint of Tequila 100% agave silver class, defined from the average values of δ^13^C_VPDB_ of the molecules of ethyl acetate, methanol, n-propanol, isoamyl alcohol, and ethanol. The dashed lines represent the acceptance range of two standard deviations.

**Figure 5:**
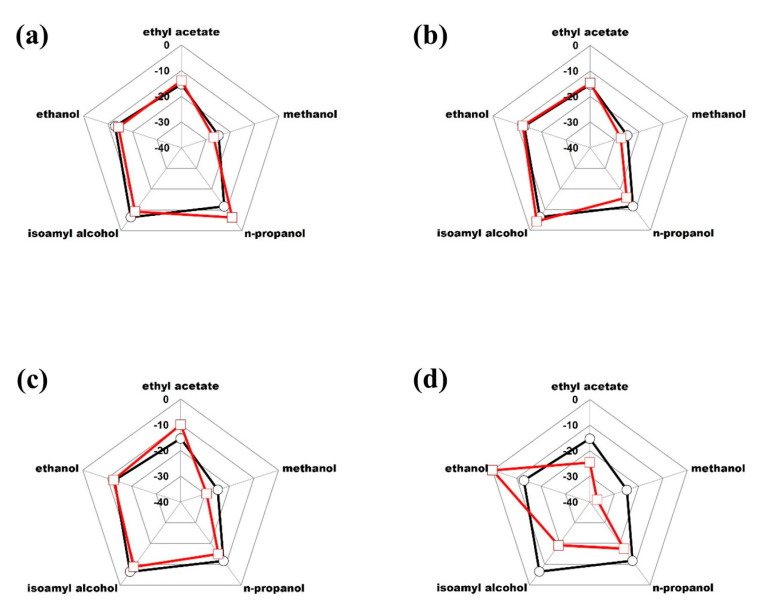
Isotopic fingerprint (radar plot) of δ^13^C_VPDB_ (‰) mean values for Tequila 100% agave silver class (black line) versus (**a**) Mezcal, (**b**) Bacanora, (**c**) Raicilla and (**d**) Sotol (red line).

The isotopic fingerprint of the beverage (Figure 4) was used to study significant differences between the Tequila 100% agave silver class against selected samples of alcoholic beverages produced from varieties of agave plants different from the *tequilana* Weber blue variety (Mezcal, Bacanora, Raicilla, and Sotol), the respective fingerprints of which are presented, for comparison, in Figure 5.

The δ^13^C_VPDB_ values were evaluated for all congeners, which do not present significative differences with respect to Tequila values (*p* > 0.05; see Supplementary Figures S2 and S3), which is to be expected due to the fact that these beverages are produced from different agave species (Mezcal: Agave *angustifolia* Haw, Agave *esperrima* Jacobi, Agave *weberi* Cela, Agave *potatorum* Zucc, and Agave *salmiana* Otto Ex Salm SSP Crassispina (Trel) Gentry; Bacanora: Agave *angustifolia* Haw; Raicilla: Agave *maximiliana*, Agave *inaequidens*, Agave *valenciana*, Agave *angustifolia*, and Agave *rhodacantha*), all of which belong to the CAM metabolism group and have the same photosynthesis process for CO_2_ fixation. Nevertheless, a particular case has been seen in ethyl acetate to compare Tequila against Sotol and Raicilla, where significant differences were seen (*p* < 0.05; see Supplementary Figure S3). From this, it is possible to conclude that with a comparison of the values of δ^13^C_VPDB_ from the ethyl acetate compound of Tequila 100% agave (average of −15.30‰), in contrast with Mezcal (average of −14.16‰), Bacanora (average of −16.47‰), Raicilla (average of −10.61‰), and Sotol (average of −21.28‰), such results are attributable to the fermentation process due to the yeast strains and conditional parameters used in its production [43]. The proposal for the δ^13^C_VPDB_ values is more negative in this congener due to the operating conditions in the fermentation stage. It has been evidenced that several operation conditions can affect the physicochemical profile of the beverages, such as pH, temperature, nutrients, and kind of yeast [11,32,42,44]; among them, the type of yeast stands out, which has been shown to be the one that has the most influence in producing this congener [45]. In such a way, the production of ethyl acetate can be related due to the type of yeast and plants used, due to the fact that there is a complex amount of nutrients that enhance fermentation according to its concentration, such as sugars, amino acids, fatty acids, and micronutrients, which impact the fermentation process.

It is important to denote a particular case that has been seen when the isotopic fingerprint of Tequila 100% agave was compared with Sotol (Figure 5d). Significant differences were appreciated (*p* < 0.05; see Supplementary Figures S2 and S3) for most congeners studied, highlighting that the value of δ^13^C_VPDB_ for ethanol (−11.95‰) is attributable to the agave plant used for the Sotol production (*Dasylirion wheeleri* agave), which presents CO_2_ due to a reaction catalyzed by the enzyme phosphoenolpyruvate carboxylase (PEP-carboxylase), and the first products from CO_2_ fixation are four-carbon dicarboxylic acids (aspartic, oxaloacetic, and malic), which can be considered as C3 plant related to δ^13^C_VPDB_ values near −10‰ [46,47].

**Figure 6:**
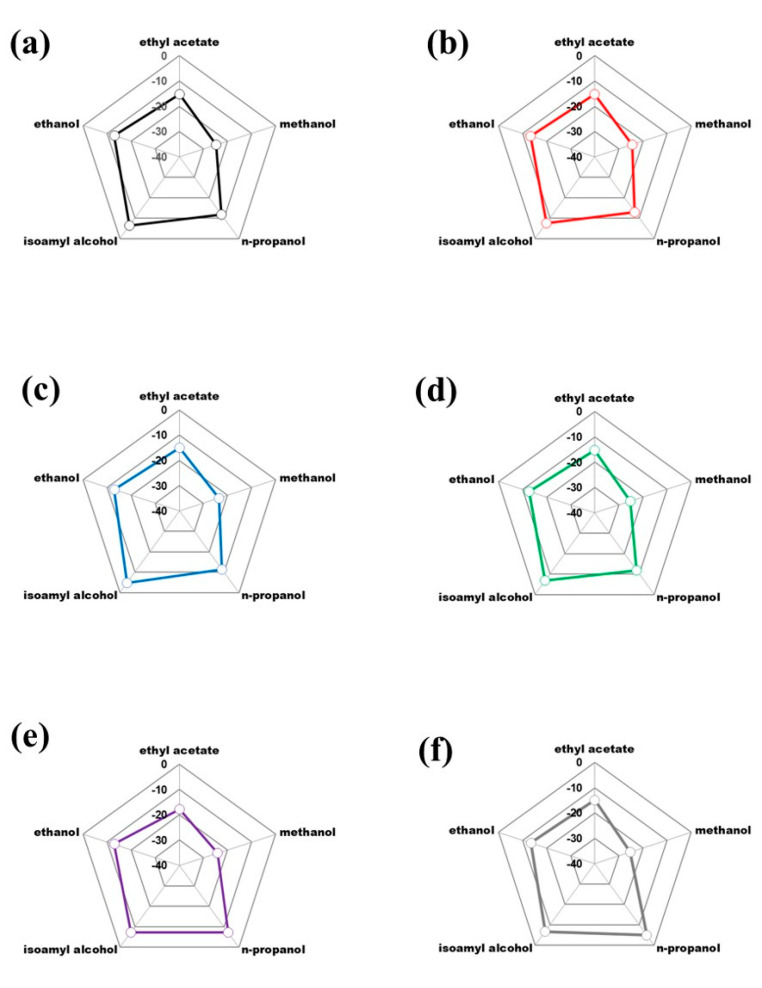
Isotopic fingerprint (radar plot) of δ^13^C_VPDB_ (‰) mean values for a Tequila 100% agave silver class according to the geographic region: (**a**) Altos sur, (**b**) Centro, (**c**) Cienega, (**d**) Valles, (**e**) Guanajuato and (**f**) Michoacán.

Finally, as part of the statistical analysis of the data, radar plots (isotopic fingerprints) were constructed for each of the analyzed regions (Figure 6); it is highlighted that there are no significant differences between regions (*p* > 0.05; see Supplementary Figures S4 and S5), evidencing that the production processes of the entire western region granted by the DOT present high-quality standards in the final product.

### 3.3. Auxiliar Diagnostic Test to Determine the Authenticity of the Beverage

To consider the above information, it is concluded that the use of a single value of δ^13^C_VPDB_ from these molecules to evaluate the authenticity of Tequila 100% agave silver class needs the combination of all of them because the physicochemical characteristic profile of this beverage is granted due to all these congeners. Although some values can give us more information to cases in the comparison between specific beverages, such as Mezcal or Bacanora, they still result insufficient as discriminants. For this reason, the design of a diagnostic test, as shown in Section 2.3, was conducted with the objective to use an auxiliar technique to determine authenticity in the beverage.

Even though the δ^13^C_VPDB_ values of ethanol, methanol, and ethyl acetate were the congeneric compounds with less variation in the Tequila 100% agave samples analyzed in the present study, the score associated with the diagnostic test was assigned with a higher number than the rest of the compounds because, as was mentioned before, these values are dependent on the type of plant used as the sugar source to produce the alcohol [23], as well as processes linked to good practices in the hydrolysis [34,48] and distillation stages [49]. For the rest of the congeneric compounds, its δ^13^C_VPDB_ values have more variability due to the fact that these are influenced principally by biochemical process that take place in the fermentation stage, where the use of different yeast strains and operational conditions can grant unique organoleptic properties. The fact that there is a variety of alcoholic beverages makes it possible to focus on a particular market [50]. For isoamyl alcohol and ethyl lactate, its presence in the physicochemical profile of Tequila has been mainly related to the fermentation process, where factors that impact this stage could be distinguished, such as yeast strain, temperature, and carbon/nitrogen ratio, which affect the alcoholic fermentation route in which compounds are synthetized [36,37]. Additionally, the presence of certain compounds, such as Maillard reactions, which are obtained from various molecules, such as amino acids or proteins, and reducing carbohydrates also had an impact [51]. Finally, the alterations observed in the δ^13^C_VPDB_ values from the molecule of n-propanol can be related to modifications in the production of this beverage, which can be by the use of raw material with less age, such as agaves from 4 to 6 years, in the Tequila production, the use of which modifies the juice/exudate ratio in the fermentation tanks, which favors a metabolic route that produces this alcohol [32]. For this reason, the score associated in the diagnostic test to these compounds is less to evaluate authenticity in the beverage.

It is important to highlight that the values of δ^13^C_VPDB_ for the rest of the congeneric compounds evaluated, in the present research work and in preliminary studies (acetaldehyde, acetal, 2-butanol, isobutanol, n-butanol, and n-pentanol), show greater variability because these are mainly related to biochemical processes in the fermentation stage, where the use of different yeasts and operating conditions will grant specific organoleptic properties. It has been shown that its presence in the beverage is due to different metabolic routes associated to the genetic variability of yeasts, and an increase or decrease in some operational parameters affects its production. Biosynthetic routes of molecules such as 2,3-butanediol, leucine, isoleucine, and pantothenate are competitive ways that regulate the synthesis of isobutanol [44,45,52,53,54]. It is for this reason that its presence in the samples of Tequila 100% agave silver class is fluctuating, so they have not been added within the isotopic fingerprint of the beverage to improve the sensitivity and specificity of the diagnostic test.

**Figure 7:**
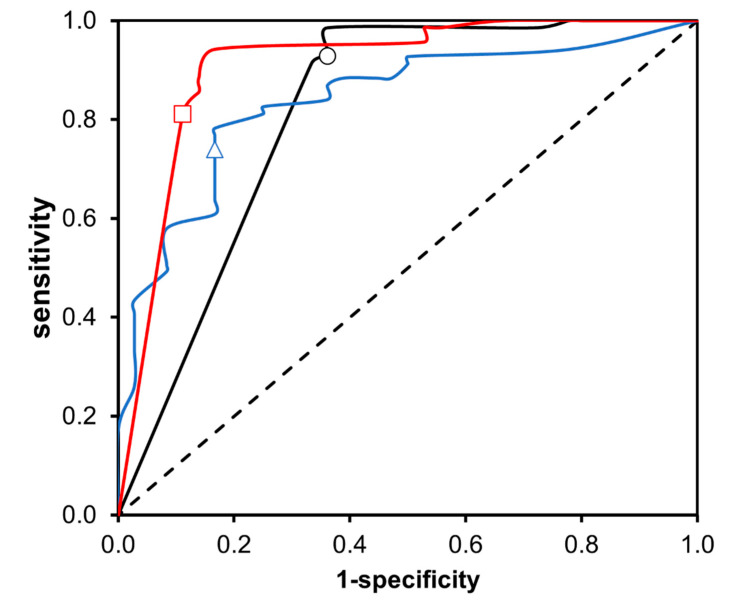
ROC curves from the diagnostic tests conducted to determine authenticity of Tequila 100% agave silver class sample. Threshold Criteria: ○ ±1s, □ ± 2s and ∆ ±3s.

Taking the above into account, the score obtained of each δ^13^C_VPDB_ value has been related to its coefficient variation presented in Table 2. The first step was to evaluate if δ^13^C_VPDB_ value was found between the limits established in ±1s, ±2s, and ±3s. In case the value was within the range, the assignment of a score was given being the C.V. value. Finally, the global score of a sample was used to determine its authenticity, establishing the multiplication of all the granted scores for each of the congeners studied. Once all samples had their respective score, the statistical analysis was conducted by employing the method of the Receiver Operating Characteristic (ROC) curve. The calibration of the diagnostic tests was carried out taking into consideration all the samples of Tequila 100% agave silver class as “Positive” and alcoholic beverages from other agave species (Mezcal, Bacanora, Raicilla and Sotol) as “Negative”. The results obtained were: Area Under Curve (AUC), Sensibility, Specificity, and Youden’s Index for each of the diagnostic tests (presented in Table 4 and Figure 7).

Figure 7 shows the ROC curves obtained. The dashed line represents a test without diagnostic discriminatory capacity, since the AUC corresponds to 0.5, so it is defined as a random classifier. The ROC curves obtained for the evaluated diagnostic tests are presented in a continuous line. The variations in the plots are because a higher value on the “1-specificity” axis indicates a greater number of false positives than true negatives, while a higher value on the “sensitivity” axis indicates a greater number of true positives than false negatives. Therefore, the choice of threshold depends on the ability to balance between false positives and false negatives. It can be concluded that the threshold criteria are in the ±2s category, maximizing the Youden´s Index (0.78), which can be defined as the best diagnostic test from the four tested, also granting a sensibility and specificity of 94.2% and 83.3%, respectively. The diagnostic test designed from the determination of δ^13^C_VPDB_ values of the congeneric compounds present in a beverage provides reliable results. However, it is important to highlight the need to increase the study for the number of samples of Mezcal, Bacanora, Raicilla and Sotol to increase the specificity of the diagnostic test, since as the number increases of experimental data analyzed, the statistics that describe them will change, improving the values of sensitivity and specificity. In addition to this, it should be noted that the analytical method and diagnostic test proposed here should not be considered unique when evaluating the authenticity of a sample of Tequila 100% agave silver class. The methods proposed here should be considered as complementary to conventional analytical techniques defined in the official standards, as well as to the current on-site inspection processes carried out by the Tequila Regulatory Council, for which the values of sensitivity (94.2%) and specificity (83.3%) obtained now can be considered suitable when combined with the rest of the tools available in the CRT to declare a beverage as authentic or non-authentic.

### 3.4. Auxiliar Diagnostic Test to Determine the Authenticity of Suspected Beverages

As mentioned in the Section 1, the current problem faced by the agave–Tequila production chain is the presence of non-authentic beverages in the market, which results in economic effects and damage to the image of the Tequila brand. To demonstrate that the analytical technique and diagnostic test developed in this research work are robust and can be used to complement the current methods proposed in the official standards, two samples were selected that have been sampled by the CRT due to the detection of inconsistencies within the inspection process. Problem sample 1 (PS1) corresponds to a sample with a high content of methanol and higher alcohols, while problem sample 2 (PS2) corresponds to a confiscated sample that meets all the parameters established in the official standard. Additional to the one previously analyzed, a non-authentic sample was prepared in the laboratory (PS3); its preparation is shown in Supplementary Table S3. The concentrations were established according to specifications that are permitted in the Official Mexican Standard NOM-006-SCFI-2012. A typical chromatogram of a sample problem is presented in Supplementary Figure S6.

Table 5 shows the results obtained from the characterization of beverages by gas chromatography, as dictated by the official standard.

**Figure 8:**
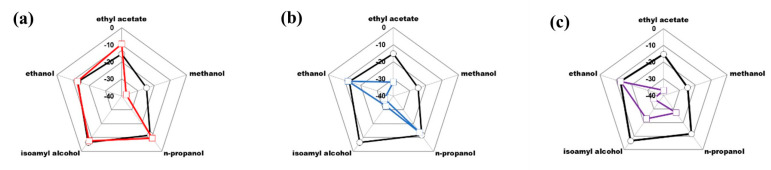
Graphic comparison of the isotopic fingerprints of problem samples against that of Tequila 100% agave silver class (black line): (**a**) problem sample 1 (PS1), (**b**) problem sample 2 (PS2), and (**c**) problem sample 3 (PS3).

The analytical method and diagnostic test designed from the determination of δ^13^C_VPDB_ of the molecules of ethyl acetate, methanol, n-propanol, isoamyl alcohol and ethanol were used. Figure 8 shows the graphic comparison of the isotopic fingerprints of the problem samples against the isotopic fingerprint of Tequila 100% agave silver class. Finally, when carrying out the mathematical calculations dictated by the diagnostic test, it is defined that in all the samples studied, the values obtained are below the authenticity criterion (0.0311), concluding that the beverages do not correspond to Tequila 100% agave silver class (Table 6).

From the chromatographic analysis, it is concluded that the PS1 sample can be classified as out of standard for exceeding the parameters established in the Official Mexican Standard NOM-006-SCFI-2012. Additionally, from the analysis of δ^13^C_VPDB_, it is concluded that the beverage can be classified as produced from other species of agave, different from the *tequilana* Weber blue variety, since the values obtained show a high similarity against that reported for beverages produced from agave *Dasylirion wheeleri* (−35.14 ‰ in average), for which the product can be classified as non-authentic. In addition, differences in methanol values can be attributed to the hydrolysis and distillation process, which is most of the time being carried out by artisanal methods. This practice can produce an enzymatic demethoxylation of lignocellulosic materials and pectins with the consequent generation of methanol during the hydrolysis process.

In the case of samples PS2 and PS3, it is concluded that they correspond to alcoholic beverages produced from ethanol of different origin by incorporating chemical reagents of synthetic origin, such as methanol, isoamyl alcohol and ethyl acetate, for which the product can be classified as non-authentic (negative in the statistical test). This was in such a way that the proposed method and diagnostic test strengthen the current methods present by official standards and the inspection processes carried out by the CRT on an ongoing basis.

## 4. Conclusions

The combination of these congeneric compounds that enrich the Tequila 100% agave silver class establishes its physicochemical profile and grants its unique organoleptic properties to the beverage. The δ^13^C_VPDB_ values for ethanol, methanol, and ethyl acetate presented less variation, which can be attributed to the fact that they belong to the plant of *Agave tequilana* Weber blue variety (CAM pathway plant). Moreover, δ^13^C_VPDB_ values for acetaldehyde, n-propanol, isobutanol, isoamyl alcohol, and ethyl lactate presented greater variation related to those different metabolic routes that are involved also because of the genetic variability in yeasts, as well as operational conditions in the Tequila companies. These characteristics give Tequila 100% agave a unique isotopic fingerprint, which can be represented in a radar plot and is useful in determining the authenticity of the beverage. This information has been useful in building a robust diagnostic test capable of discriminating between Tequila 100% agave silver class, non-authentic beverages, and alcoholic beverages produced from other species of agave different from *tequilana* Weber blue variety.

## Figures and Tables

**Table 1:** Stratified analysis by proportional allocation used for the analysis of Tequila 100% agave silver class.

Region	Total Number of Tequila Producers Active in the Region	%	Samples	%
Valles (Jalisco)	62	42	28	41
Altos Sur (Jalisco)	46	31	21	31
Cienega (Jalisco)	21	14	10	14
Centro (Jalisco)	14	9	7	10
Guanajuato	4	3	2	3
Michoacan	2	1	1	1
Total	149	100	69	100

**Table 2:** Statistical analysis of the data obtained for the analyzed samples of Tequila 100% agave silver class.

Statistic	Acetaldehyde	Ethyl Acetate	Methanol	n-Propanol	Isoamyl Alcohol	Ethyl Lactate	Ethanol
	(‰)	(‰)	(‰)	(‰)	(‰)	(‰)	(‰)
$\mathrm{Average}\;(\bar{X}$)	−15.7	−15.3	−24.8	−11.7	−6.5	−10.7	−12.9
Standard deviation (s)	4.2	1.5	1.0	4.1	1.6	2.6	0.4
−2s	−24.02	−18.32	−26.82	−19.89	−9.63	−15.98	−13.76
+2s	−7.40	−12.28	−22.80	−3.47	−3.33	−5.39	−12.09
Lower quartile (Q_L_)	−18.63	−16.23	−25.45	−14.23	−7.46	−11.39	−13.23
Upper Quartile (Q_U_)	−13.53	−14.22	−24.13	−8.88	−5.40	−9.38	−12.67
Median ($\tilde{X}$)	−15.85	−15.49	−24.78	−10.48	−6.55	−10.38	−12.94
Coefficient of Variation (C.V.)	−0.26	−0.10	−0.04	−0.35	−0.24	−0.25	−0.03
number of observations	63	67	69	68	69	65	69
Minimum value (min)	−23.60	−18.82	−27.71	−21.77	−11.76	−23.73	−13.78
Maximum value (max)	−5.39	−10.22	−22.99	−4.84	−3.51	−4.41	−11.34

**Table 3:** Fisher’s linear discriminant analysis structure matrix.

Molecule	Function		
	1	2	3
Ethyl acetate	0.717	0.211	−0.052
Methanol	−0.415	0.220	0.363
Isoamyl alcohol	−0.024	−0.635	0.633
Ethanol	0.160	0.479	0.102
Isobutanol	0.005	−0.336	−0.050
Acetaldehyde	0.174	0.174	0.429
n-propanol	0.381	0.155	0.760
Ethyl lactate	0.017	−0.064	−0.308

**Table 4:** Receiver Operating Characteristics parameters obtained by evaluating physicochemical properties in Tequila 100% agave silver class and other agave alcoholic beverages produced from different species of agave.

Threshold Criteria	AUC *	Standard Error (%)	Sensibility (%)	Specificity (%)	Youden’s Index	Positive if the Test Value Is Greater Than
±1s	0.81	5.1	98.6	63.9	0.62	0.0311
±2s	0.91	3.6	94.2	83.3	0.78	0.0311
±3s	0.84	4.0	78.3	83.3	0.62	0.0024

* AUC: Area under the Curve.

**Table 5:** Results of the gas chromatography tests of the problem samples.

Congeners (mg/100 mL A.A. *)	Problem Sample 1 (PS1)	Problem Sample 2 (PS2)	Problem Sample 3 (PS3)	Limits Established by the Official Mexican Standard NOM-006-SCFI-2012
Methanol	2350.6	137.1	144.13	30–300
Higher alcohols	14,548.6	244.7	312.88	20–500
Esters	180.3	40.3	60.63	2–200
Aldehydes	20.3	16.5	23.38	0–40

* Anhydrous alcohol.

**Table 6:** Result of the analysis of the diagnostic test in non-authentic alcoholic beverages and an alcoholic beverage made from cane ethanol.

Sample	Ethyl Acetate (‰)	Methanol (‰)	n-Propanol (‰)	Isoamyl Alcohol (‰)	Ethanol (‰)	Test Value ±2s	Positive if the Test Value Is Greater Than:	Test ±2s
PS1	−9.48	−37.22	−9.43	−7.53	−12.88	0.0018	0.0311	Negative
PS2	−31.89	−45.45	−13.77	−32.66	−12.15	0.0006	0.0311	Negative
PS3	−36.92	−46.18	−27.23	−22.68	−13.08	0.0003	0.0311	Negative
Reference values for Tequila 100% agave silver class *	−13.8 to −16.8	−23.8 to −25.8	−7.6 to −15.8	−4.9 to −8.1	−12.5 to −13.3	---	---	---

* Values obtained in this study.

## Data Availability

The data that support the findings of this study are available from the corresponding author upon reasonable request.

## Supplementary Materials

The following supporting information can be downloaded at: https://www.mdpi.com/article/10.3390/foods12132605/s1, Table S1: Operating conditions of the GC/C/IRMS analyses; Table S2: Type of raw material used and region that delimits the appellation of origin of alcoholic beverages produced from different species of agave; Table S3: List of reagents used for PS3 sample preparation; Figure S1: Typical GC/C/IRMS chromatogram of a sample of Tequila 100% agave Silver class; Figure S2: Statistical analysis (ANOVA) of the δ^13^C_VPDB_ values for the molecules of (a) acetaldehyde, (b) ethyl acetate and (c) ethyl lactate in different alcoholic beverages produced from agave plants; Figure S3: Statistical analysis (ANOVA) of the δ^13^C_VPDB_ values for the molecules of (a) methanol, (b) ethanol, (c) n-propanol and (d) isoamyl alcohol in different alcoholic beverages produced from agave plants; Figure S4: Statistical analysis (ANOVA) of the δ^13^C_VPDB_ values for the molecules of (a) acetaldehyde, (b) ethyl acetate and (c) ethyl lactate in samples of Tequila 100% agave silver class produced in different regions of the DOT. Figure S5: Statistical analysis (ANOVA) of the δ^13^C_VPDB_ values for the molecules of (a) methanol, (b) ethanol, (c) n-propanol and (d) isoamyl alcohol in samples of Tequila 100% agave silver class produced in different regions of the DOT; Figure S6: Typical chromatogram of a sample problem (identification: PS2).
