# Supplementary material for: Design of a Novel Auxiliary Diagnostic Test for the Determination of Authenticity of Tequila 100% Agave Silver Class Based on Chemometrics Analysis of the Isotopic Fingerprint of the Beverage
Source: Foods. 2023 Jul 5;12(13):2605. doi: 10.3390/foods12132605 (PMC10341325; doi:10.3390/foods12132605)
Supplement: Supplementary file 1 [file foods-12-02605-s001.zip › foods-2471079-supplementary.pdf]

foods

MDPI

Article

Design of a novel auxiliary diagnostic test for the determination of authenticity of Tequila 100% agave silver class based on chemometrics analysis of the isotopic fingerprint of the beverage

Rocío Fonseca-Aguiñaga[1,2], Uriel E. Navarro-Arteaga[1,2], Martin Muñoz-Sánchez[3], Humberto Gómez-Ruiz[4], Walter M. Warren-Vega[1], Luis A. Romero-Cano[1,*]

1 Grupo de Investigación en Materiales y Fenómenos de Superficie, Departamento de Biotecnológicas y Ambientales, Universidad Autónoma de Guadalajara, Av. Patria 1201, Zapopan, Jalisco C.P. 45129, Mexico
2 Laboratorio de Isotopía, Consejo Regulador del Tequila A. C. Av. Patria 723, Zapopan, Jalisco C.P. 45030, México
3 Consejo Regulador del Tequila A. C. Av. Patria 723, Zapopan, Jalisco C.P. 45030, Mexico
4 Facultad de Química, Departamento de Química Analítica. Universidad Nacional Autónoma de México. Ciudad de México, C.P. 04510, Mexico
* Correspondence: luis.cano@edu.uag.mx

Supplementary Material

Table S1. Operating conditions of the GC/C/IRMS analyses.

Instrumentation	($\delta^{13}C$) GC/C/IRMS
Gas chromatograph	Thermo, Model: Trace 1310 with capillary injection system
Autosampler	Thermo, Model: Triplus RSH
Syringe	Thermo Fisher Scientific, Syringe 10 μl x 50 mm Needle Cone. P/N: 365D0291

Interfaces	Thermo Scientific model Conflo IV, continuous flow coupled to the massspectrometer of isotopic ratios.
Mode	Continuous Flow, Configuration CO_2
Oven	Maximum temperature of 450 °C
Column	DB-Wax column.<br>Stationary phase of polyethylene glycol.<br>60 m long, 0.25 mm internal diameter and 0.25 mm thick.
	**Chromatographic conditions**
Carrier gas	Helium, constant flow, 1.2 mL min^{-1}
Temperature	37.0 °C during 3.5 min, 4.0 °C min^{-1} until 78.0 °C during 1.5 min, 0.5 °C min^{-1} until 80.0 °C during 1.0 min, 15.0 °C min^{-1} until 160.0 °C during 3.0 min, 50.0 °C min^{-1} until 220.0 °C during 2.5 min, total 33 min
Injector	Mode: Split, Relation Split:<br>1:40, 200 °C
Other gases	Oxygen to regenerate thecombustion reactor
Reactor temperature	960 °C
Injection volume	1.0 μl

List of reagents and gases for the systems utilized during the analytical determinations

Reagents

- Secondary ethanol standard, traceable to the IAEA identified as BCR-656 with a value of $\delta^{13}C_{VPDB}$ of -26.91 ‰.
- Secondary standard of ethanol (from cane origin) identified as Alcohol V with a value of $\delta^{13}C_{VPDB}$ of -13.32 ‰.
- Secondary standard of ethanol (from grape origin) identified as 067I with a value of $\delta^{13}C_{VPDB}$ of -26.235 ‰.
- Standard prepared in the CRT laboratory known as Control Sample (Tequila 100% agave) with a value of $\delta^{13}C_{VPDB}$ of -13.01 ‰.
- Standard from the proficiency test identified as 838I with a value of $^{13}C_{VPDB}$ of -24.97 ‰.

Gases for the systems GC/C/IRMS

- Helium (99.999% purity), used as a carrier gas in the chromatograph and in the Isolink GC; as dilution gas in Conflo IV.
- Carbon dioxide (99.998% purity), used as reference or working gas during the On-Off Standard exercises.
- Oxygen (99.999% purity), used as a regenerating gas of the combustion reactor.
- Carbon dioxide with a known value of $\delta^{13}C$, to normalize the carbon dioxide tank with 99.998% purity, used as a working gas (or reference).

Table S2. Type of raw material used and region that delimits the appellation of origin of alcoholic beverages produced from different species of agave

Beverage	Region of Denomination of Origin	Raw Material	Official standard
Tequila	Jalisco (125 municipalities), Nayarit (8 municipalities), Michoacán (30 municipalities), Guanajuato (7 municipalities), Tamaulipas (11 municipalities)	*Agave tequilana* Weber variedad Azul	NORMA OFICIAL MEXICANA NOM-006-SCFI-2012, BEBIDAS ALCOHÓLICAS-TEQUILA-ESPECIFICACIONES
Mezcal	Oaxaca (570 municipalities), Durango (39 municipalities), Guanajuato (2 municipalities), Guerrero (81 municipalities), Michoacán (29 municipalities), San Luis Potosí (58 municipalities), Puebla (116 municipalities), Tamaulipas (11 municipalities), Zacatecas (58 municipalities), Aguascalientes (7 municipalities), Morelos (23 municipalities), Estado de México (15 municipalities) and Sinaloa (4 municipalities)	*Agave angustifolia* Haw<br>*Agave esperrima* Jacobi, Amarilidáceas<br>*Agave weberi* Cela, Amarilidáceas<br>*Agave potatorum* Zucc, Amarilidáceas<br>Agave s*almiana* Otto Ex Salm SSP Crassispina (Trel) Gentry<br><br>Other species of agave, as long as they are not used as raw material for other beverages with appellations of origin within the same state	NORMA OFICIAL MEXICANA NOM-070-SCFI-2016 BEBIDAS ALCOHÓLICAS-MEZCAL-ESPECIFICACIONES
Sotol	Durango, Coahuila y Chihuahua	*Agave dasylirion* Wheeleri,	NORMA OFICIAL MEXICANA NOM-159-SCFI-2004, BEBIDAS ALCOHOLICAS-SOTOL-ESPECIFICACIONES
Bacanora	Sonora (35 municipalities)	*Agave* a*ngustifolia* Haw	NORMA OFICIAL MEXICANA NOM-168-SCFI-2005, BEBIDAS ALCOHÓLICAS-

			BACANORA-ESPECIFICACIONES DE ELABORACIÓN, ENVASADO Y ETIQUETADO
Raicilla	Jalisco (16 municipalities), Nayarit (1 municipality)	*Agave maximiliana* Baker<br>*Agave inaequidens* Koch<br>*Agave valenciana*<br>*Agave angustifolia* Haw<br>*Agave rhodacantha*<br><br>Other species of agave, as long as they are not used as raw material for other beverages with appellations of origin within the same state	DECLARACIÓN GENERAL DE PROTECCIÓN DE LA DENOMINACIÓN DE ORIGEN RAICILLA.

Table S3. List of reagents used for PS3 sample preparation

Reagent	Supplier	Purity	Volume used (mL)
Sugar cane alcohol	Alcoholera de Zapopan	96° G.L:	573.00
Acetaldehyde	Merck	99%	0.13
Ethyl acetate	Sigma Aldrich	≥ 99.5%	0.61
Methanol	Sigma Aldrich	≥ 99.8%	1.10
n-propanol	Sigma Aldrich	≥ 99%	0.86
Isobutanol	Sigma Aldrich	≥ 99%	0.86
Isoamyl alcohol	Merck	99%	0.85
Ethyl lactate	Sigma Aldrich	98%	0.27

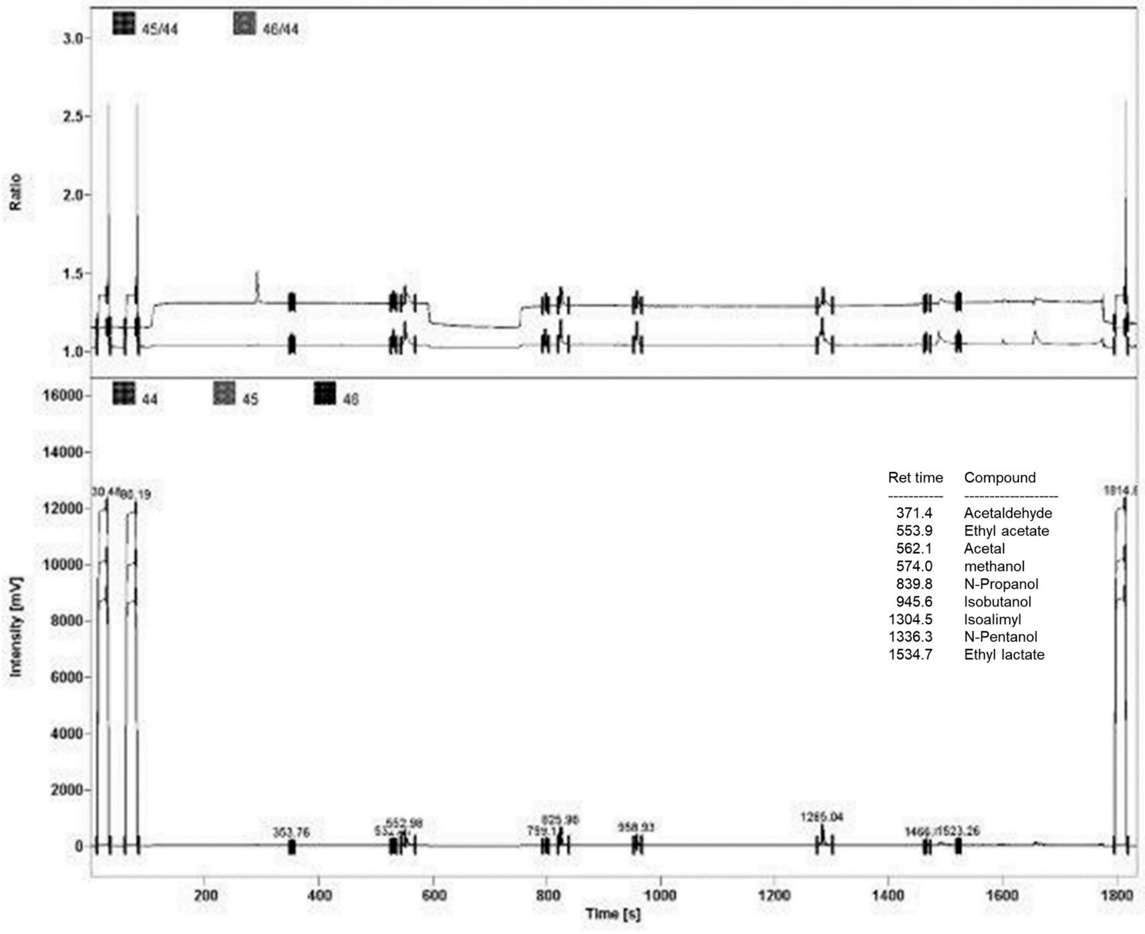

Figure S1. Typical GC/C/IRMS chromatogram of a sample of Tequila 100% agave Silver Class

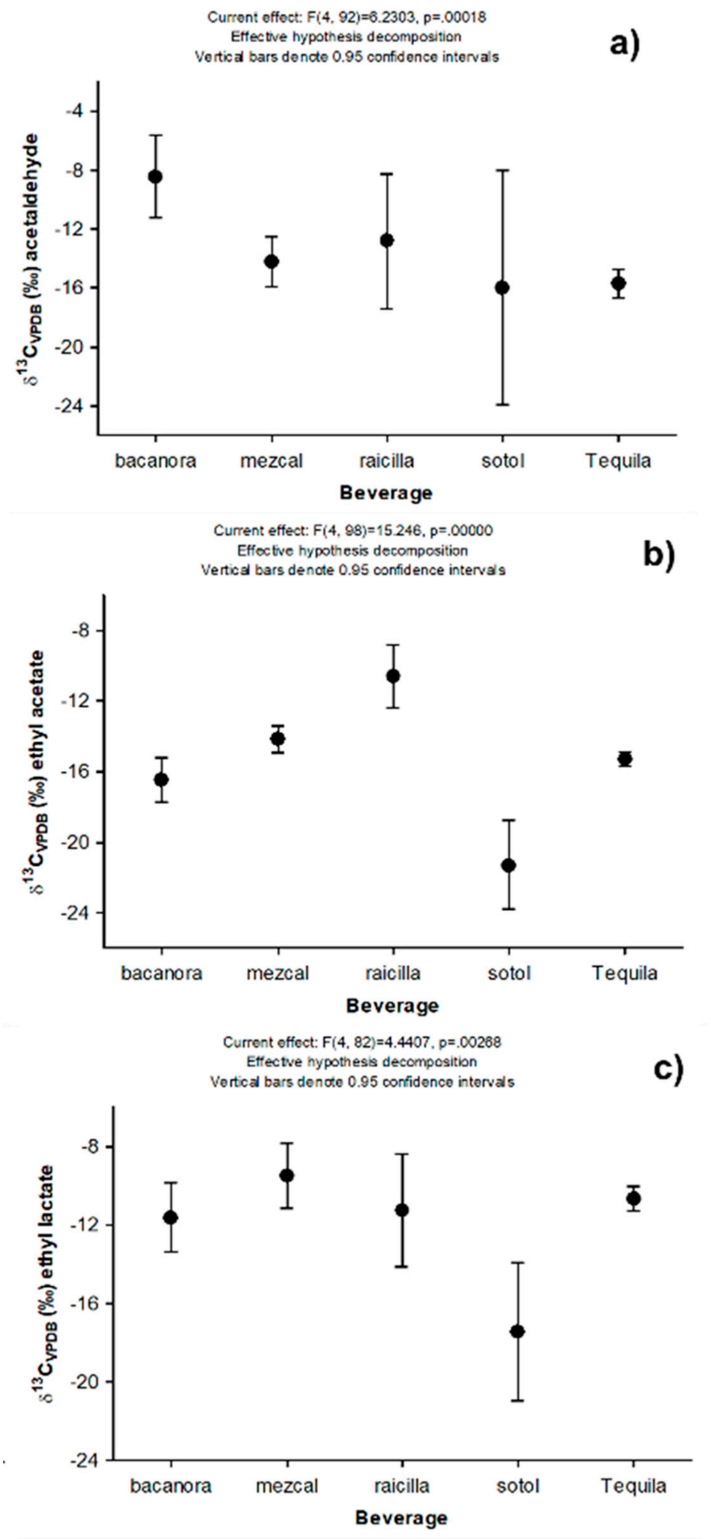

Figure S2. Statistical analysis (ANOVA) of the $\delta^{13}C_{VPDB}$ values for the molecules of (a) acetaldehyde, (b) ethyl acetate and (c) ethyl lactate in different alcoholic beverages produced from agave plants

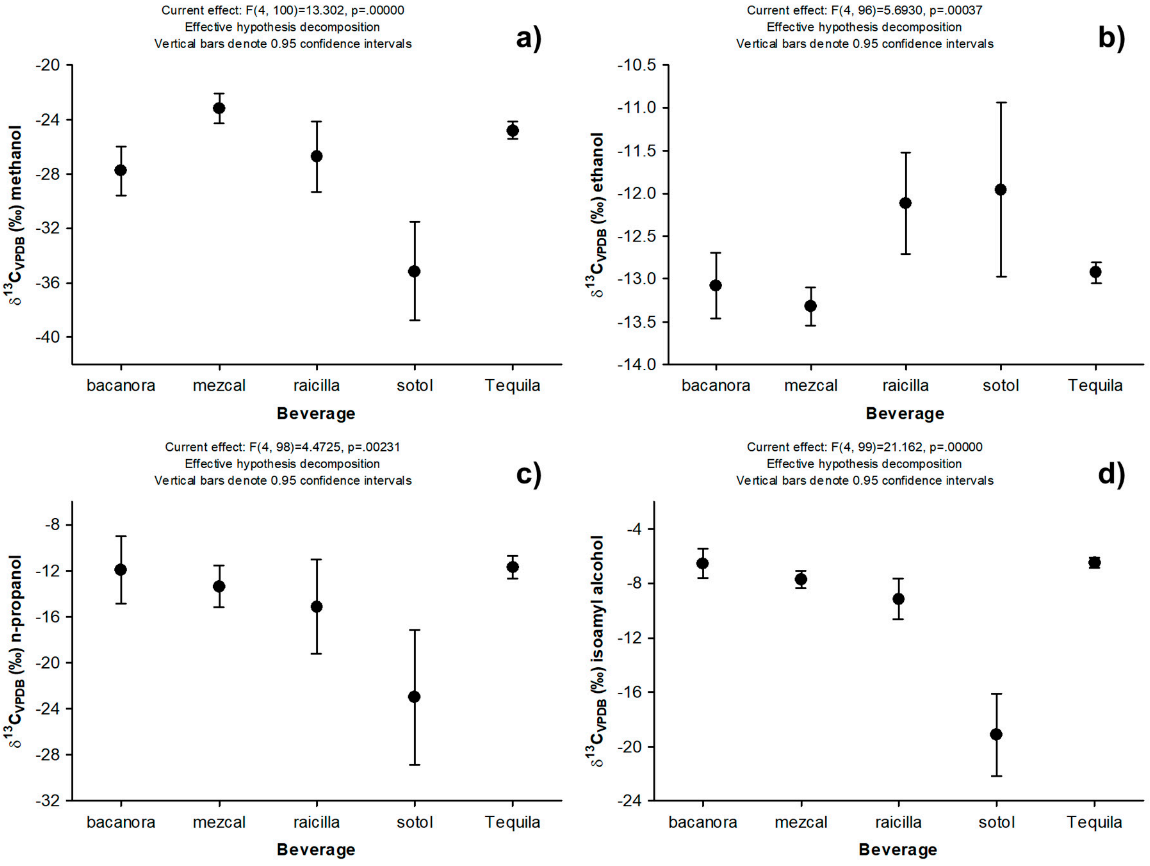

Figure S3. Statistical analysis (ANOVA) of the $\delta^{13}C_{VPDB}$ values for the molecules of (a) methanol, (b) ethanol, (c) n-propanol, and (d) isoamyl alcohol in different alcoholic beverages produced from agave plants

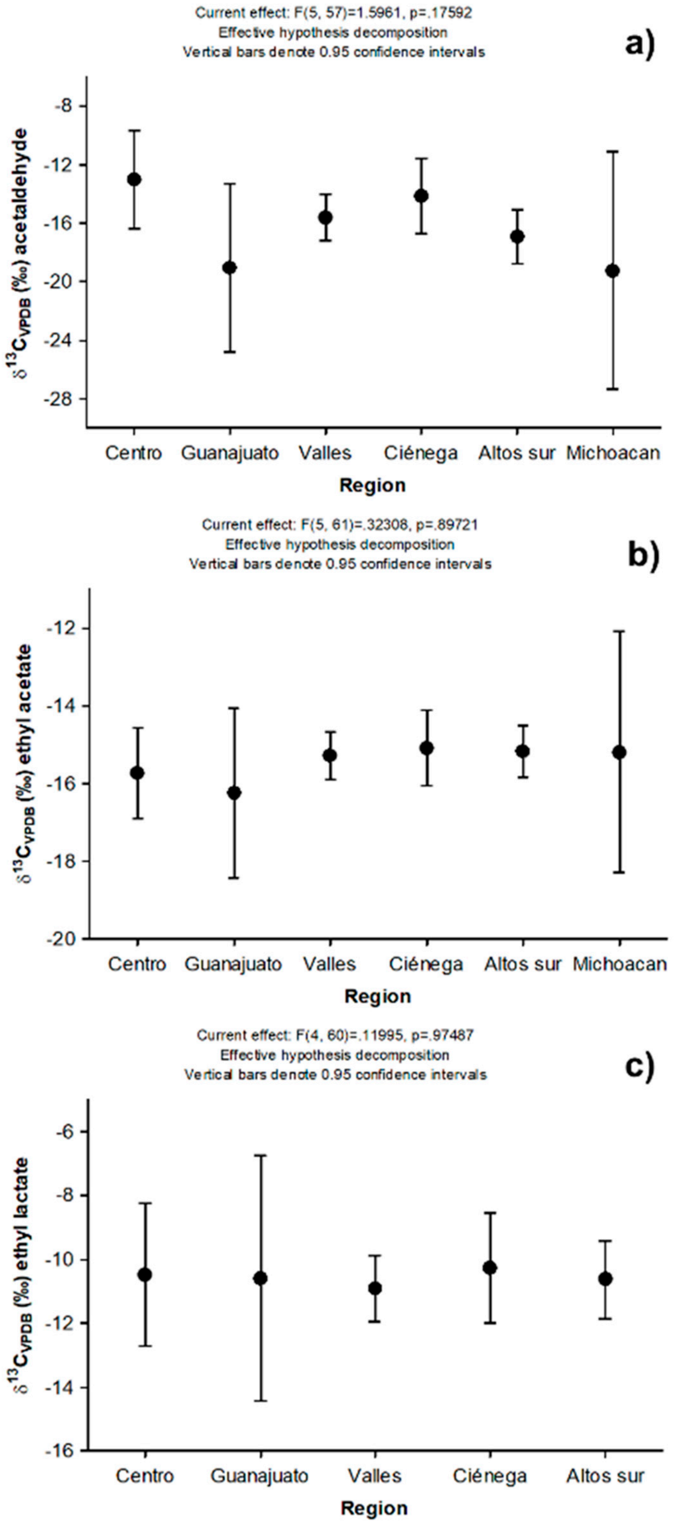

Figure S4. Statistical analysis (ANOVA) of the $\delta^{13}C_{VPDB}$ values for the molecules of (a) acetaldehyde, (b) ethyl acetate and (c) ethyl lactate in samples of Tequila 100% agave silver class produced in different regions of the DOT.

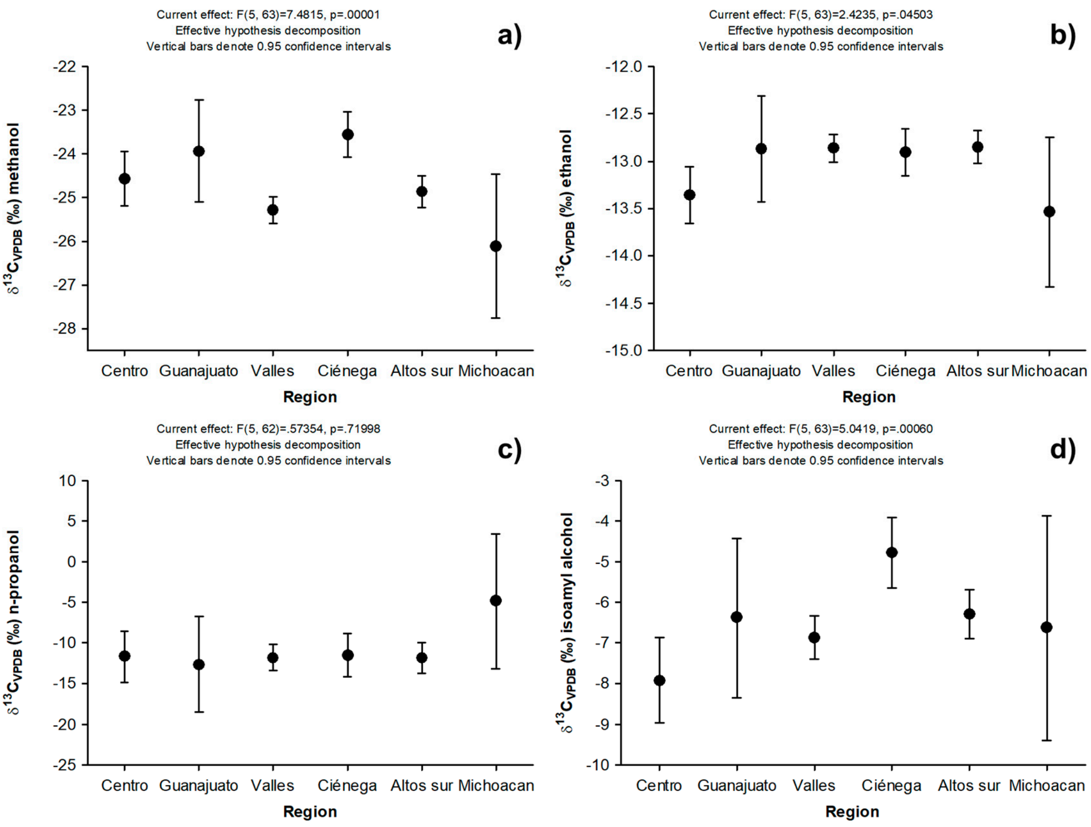

Figure S5. Statistical analysis (ANOVA) of the $\delta^{13}C_{VPDB}$ values for the molecules of (a) methanol, (b) ethanol, (c) n-propanol, and (d) isoamyl alcohol in samples of Tequila 100% agave silver class produced in different regions of the DOT.

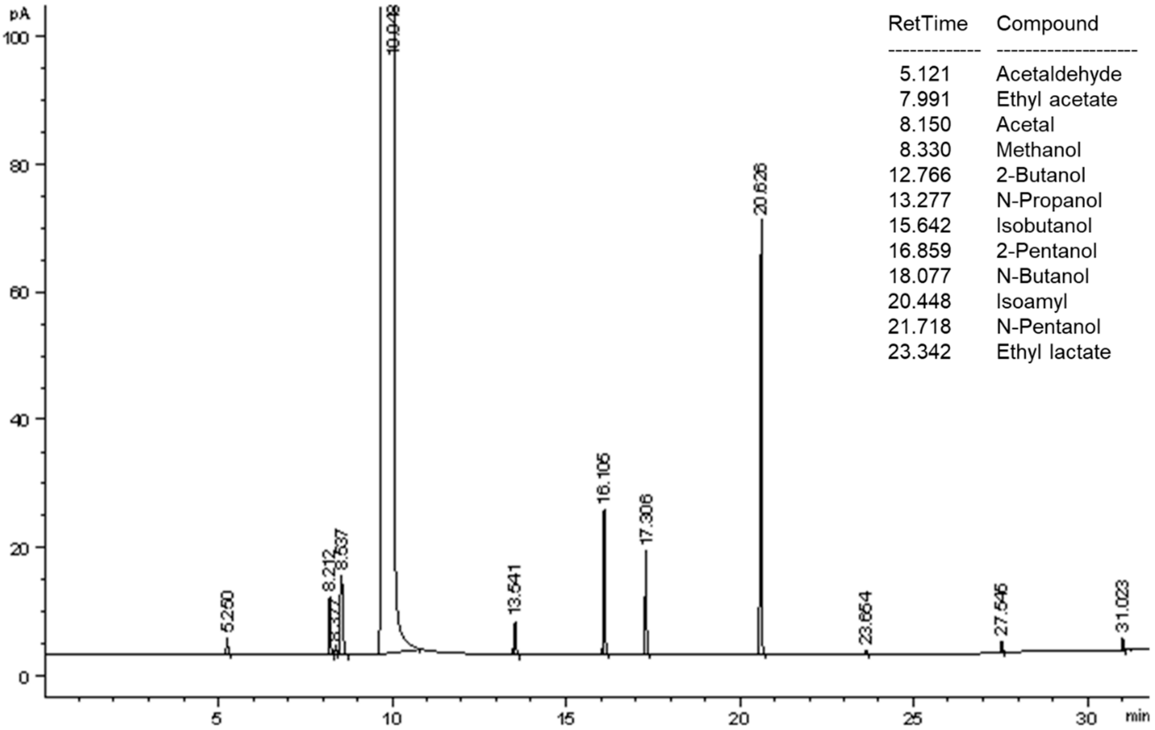

Figure S6. Typical chromatogram of a sample problem (identification: PS2)
